# Supplementary material for: Sunflower and Palm Kernel Meal Present Bioaccessible Compounds after Digestion with Antioxidant Activity
Source: Foods. 2023 Sep 1;12(17):3283. doi: 10.3390/foods12173283 (PMC10486993; doi:10.3390/foods12173283)
Supplement: Supplementary file 1 [file foods-12-03283-s001.zip › foods-2549256-supplementary.pdf]

Table S1: List of the main chemicals used in analysis.

Chemicals / Reagents	Brand	Analysis
α-amylase from porcine pancreas A3176	Sigma-Aldrich [1]	Simulated gastrointestinal digestion
Pepsin from porcine gastric mucosa P7000	Sigma-Aldrich [1]	Simulated gastrointestinal digestion
Pancreatin from porcine P1750	Sigma-Aldrich [1]	Simulated gastrointestinal digestion
Porcine bile extract B8631	Sigma-Aldrich [1]	Simulated gastrointestinal digestion
KCl	Synth [2]	Simulated gastrointestinal digestion
KH_2PO_4	Synth [2]	Simulated gastrointestinal digestion
$NaHCO_3$	Synth [2]	Simulated gastrointestinal digestion
NaCl	Synth [2]	Simulated gastrointestinal digestion / Mw distribution
$MgCl_2(H_2O)_6$	Synth [2]	Simulated gastrointestinal digestion
$(NH_4)_2CO_3$	Synth [2]	Simulated gastrointestinal digestion
NaOH	Synth [2]	Simulated gastrointestinal digestion
HCl	Synth [2]	Simulated gastrointestinal digestion
$CaCl_2(H_2O)_2$	Synth [2]	Simulated gastrointestinal digestion
$NaC_2H_3O_2.3H_2O$	Synth [2]	Simulated gastrointestinal digestion
$MgSO_4.7H_2O$	Synth [2]	Simulated gastrointestinal digestion
2,2'-Azobis(2-amidinopropane)-dihydrochloride granular (AAPH)	Sigma-Aldrich [1]	ORAC / DNA protective capacity
Fluorescein sodium salt	Sigma-Aldrich [1]	ORAC
2,2-azinobis(3-ethylbenzothiazoline-6-sulfonic acid (ABTS),	Sigma-Aldrich [1]	ABTS
2,2-difenil-1-picrilidrazil (DPPH),	Sigma-Aldrich [1]	DPPH
6-hidroxi-2,5,7,8-tetrametilcroman-2-carboxilic acid (Trolox),	Sigma-Aldrich [1]	ORAC / ABTS / DPPH
Gallic acid	Sigma-Aldrich [1]	Folin-Ciocalteau method
SYBR safe	Thermo Scientific [3]	DNA Protective capacity
$((NH_4)_2C_6H_6O_7$	Dinâmica [4]	Potential prebiotic effect
$MnSO_4.H_2O$	Dinâmica [4]	Potential prebiotic effect
K_2HPO_4	Dinâmica [4]	Potential prebiotic effect
Agar	KASVI [5]	Potential prebiotic effect
Yeast extract	KASVI [5]	Potential prebiotic effect
Beef extract	KASVI [5]	Potential prebiotic effect

Peptone bacteriological	KASVI [5]	Potential prebiotic effect
Peptic digest of animal tissue	Neogen [6]	Potential prebiotic effect
Peptone A	Neogen [6]	Potential prebiotic effect
Folin-ciocalteu reagent	Synth [2]	Folin-Ciocalteau method
n-hexane	Synth [2]	Flour defatting process
Ethanol	Synth [2]	Sample extract preparation
Methanol	Synth [2]	Sample extract preparation
Polysorbate 80	Neogen [6]	Potential prebiotic effect
Ultrapure agarose	Thermo Scientific [3]	DNA Protective capacity
2,2,2-trichloroacetic acid (TCA)	Synth [2]	Folin-Ciocalteau method
Amino acids standard	Thermo Scientific [3]	Amino acid profile
DL-2-aminobutyric acid	Sigma-Aldrich [1]	Amino acid profile
Sodium phosphate monobasic (NaH_2PO_4)	Synth [2]	Mw distribution
Sodium phosphate dibasic (Na_2HPO_4)	Synth [2]	Mw distribution
EDTA disodium salt	Synth [2]	DNA protective capacity
TRIS base, $NH_2C(CH_2OH)_3$	KASVI [5]	DNA protective capacity
α-lactalbumin (L6010)	Sigma-Aldrich [1]	Mw distribution
Insulin (I2643)	Sigma-Aldrich [1]	Mw distribution
Vitamin B12 (Fw13554)	Sigma-Aldrich [1]	Mw distribution
L-β-4-dihydroxyphenylanine (D-9628)	Sigma-Aldrich [1]	Mw distribution
Acetic Acid	Qhemis [7]	DNA protective capacity

1: Sigma Chemical Co. (St. Louis, MO, USA); 2: Synth (Diadema, SP, BRA) 3: Thermo Scientific (Rockford, IL, USA) 4: Dinâmica (Indaiatuba, SP, BRA), 5: KASVI (São José dos Pinhais, PR, BRA), 6: Neogen (Heywood, Lancashire, UK), 7: Qhemis (Jundiaí, SP, BRA)
